# Capacity of health facilities to diagnose and manage keratoconus: a Kilimanjaro region case study

**DOI:** 10.1007/s10792-025-03653-9

**Published:** 2025-08-04

**Authors:** Focus P. Maro, Vanessa R. Moodley

**Affiliations:** 1 Discipline of Optometry, School of Health Sciences, University of KwaZulu-Natal, Durban, South Africa; 2 Department of Optometry and Rehabilitation Science, Kilimanjaro Christian Medical College, P.O. Box 2240, Moshi, Tanzania; 3 Keratoconus Foundation of South Africa, PO Box 19626, Dormerton, 4015 South Africa

**Keywords:** Keratoconus, Diagnosis, Health facility, Kilimanjaro, Optometrist, Ophthalmologist

## Abstract

**Introduction:**

Keratoconus (KC), is a corneal disease that causes visual impairment, which diminishes thequality of life (QoL) of affected individuals. Although most eye care practitioners in Tanzania anecdotally report a significant number of patients presenting with KC symptoms,little is known on how KC is managed at differentlevels of the healthcare system.

**Purpose:**

To conduct a survey to assess health facility capacity and optometrists’ capability to diagnose and manage KC in Kilimanjaro region, Tanzania.

**Methods:**

A mixed-method, cross-sectional study was conducted. Health facilities providing eye care services and their employed optometristswere purposivelyselected. TheWHO Service Availability and Readiness Assessment (SARA) tool was applied to obtain eye health care service delivery data and in-depth interviews were conducted to investigate optometrists’ knowledge on the diagnosis and management modalities of KC at their respective facilities.

**Results:**

Nine multi-level health facilitieswere included in the SARA assessment.The majority of SARA respondents were female (56%) and ten optometrists participated in in-depth interviews. Only 44% of facilities were both ready to provide and had KC services available. Although all optometrists reported being aware of KC, some did not know how to comprehensively diagnose and manage KC. Barriers to optimal patient care were a lack of equipment and supplies and no practitioner training on KC.

**Conclusion:**

Findings indicate a lack of KC service availability and/or inadequate service readiness in the majority of health facilities in Kilimanjaro. Recommended remedies include general KC advocacy and a health leadership intervention to remedy reported facility and human resource deficiencies towards improved KC patient care.

## Introduction

Keratoconus (KC), a disease causing progressive corneal thinning, manifests at a young age and severelyimpacts the quality of life (QoL) of the affected individual. It is characterized by stromal thinning and steepening, which results in high myopia and irregular astigmatism, leading to distortion and subsequent vision loss [1].

The reported prevalence of KC varies with geographic location and criteria used for diagnosis. The global prevalence of KC in the general population ranges from 0.2 to 2.3% [2], while in some countries like Japan, U.K. and Russia figures are as low as 1/2000 [3, 4]. Higher population-based prevalence of KC has been reported as 4 patients per 100,000 in Saudi Arabia [5, 6]. There is a scarcity of population-based KC prevalence studies in Africa with most prevalence studies being hospital-based [7, 8]. The few population-based studies have reported a prevalence of 1.7% in Kenya [9] and 0.17% in Egypt [10]. The varied prevalence reported across countries globally has been attributed to demographic and environmental variations, making country specific data a necessary requirement for planning of KC services [11–13].

Onset varies between the early teenage years and young adulthood, seldom appearing after age35 years [15]. Meyer et al. [16], found the most frequent age ofonset to be 18 years [16], yet, the historically reported age at diagnosis ranges between 20.0 ± 6.4 and 24.05 ± 8.97 years [17, 18]. Several studies have reported delayed diagnosis of KC cases [19, 20], which may be attributed to the asymmetry of the disease, resulting in it being asymptomatic until vision is reduced in both eyes [1, 21, 22].Additionally, in many low- and medium-income countries (LMICs), poor access to eye care services deny early disease detection. The most common contributing factors of KC reported include eye rubbing, atopy, a family history of KC, warm climates and parental consanguinity, [1, 23].

### Diagnosis of KC

Keratoconus can easily be diagnosed by the scissor movement observed during retinoscopy as well as by distortion of keratometric mires or topographic images [24]. Corneal topography, which has become the gold standard method to diagnose and monitor KC, is the most sensitive method of detecting and confirming a diagnosis of KC.[25, 26]. Corneal thickness measurement by optical coherence tomography (OCT) is reported to be equally sensitive and specific as topographic indices [27, 28].

### Management of keratoconus

Treatment involves several treatment approaches to ameliorate visual compromise caused by KC, which varies depending on the stage of the condition. In the early phases, glasses or soft contact lenses are typically sufficient to manage the symptoms. As the disease advances to a moderate level, rigid gas permeable (RGP) corneal and scleral contact lenses are often prescribed to optimize visual correction [29]. In more severe cases, about 20% of patients find that contact lenses no longer offer adequate vision correction, making surgical procedures like penetrating keratoplasty necessary [22, 32]. Corneal collagen cross-linking (CXL) has been introduced, and shown promising results, in improving outcomes and slowing the progression of keratoconus [30, 31].

### Diagnosis and management of KC in Tanzania

Currently, Tanzania does not have formal guidelines for the management of KC. Although the revised guideline on staffing levels for the Ministry of Health [32] states that a primary health centre should have an optometrist, few if any, have this cadre of health care worker. Therefore, diagnosis and treatment of KC is mostly conducted starting at secondary (council/district) level and above.

At the secondary level of healthcare, the healthcare team typically includes an ophthalmologist or an assistant medical officer in ophthalmology (AMOO), an optometrist, and either a general or ophthalmic nurse. Keratoconus patients are initially seen at these secondary facilities, where the optometrist plays a pivotal role in the non-surgical treatment of KC. The optometrist can perform comprehensive vision and ocular health examinations and prescribe spectacles, if the patient's vision improves with lenses. Common diagnostic tests available at this healthcare facility level include visual acuity tests, slit-lamp examination, retinoscopy, refraction, and, if available, autorefractometry. If an ophthalmologist or AMOO is present, they may further assess the patient and either recommend glasses or contact lenses, or refer the patient to a tertiary care facility.

At the tertiary level, more advanced KC management is delivered by a specialised team, including corneal specialists, general ophthalmologists, optometrists with addition qualification of low vision and ophthalmic nurses. These health facilities offer additional diagnostic tests such as autokerato-refractometry, pachymetry, topography and tomography, with treatment options such as astigmatic spectacle lenses, rigid gas permeable (RGP) and scleral contact lenses, corneal cross-linking and keratoplasty procedures being available. Most patients present at this level in the advanced stages of the disease, mainly due to the lack of early diagnosis and limited treatment options at lower-level facilities.

Key to the delivery of health care services is an appropriately equipped facility with a knowledgeable and skilled human resource compliment. A South African study, evaluating the knowledge, skills and practices related to the diagnosis and management of KC among public sector optometrists, found that a significant proportion did not have the appropriate knowledge and skills to diagnose and manage KC patients [33]. A similar study,exploring the barriers to effective diagnosis and management of KC among Kenyan optometrists, reported that only a few were confident in the use of the keratometer and corneal topographer and had skills in the fitting of rigid gas permeable contact lenses [34].

Most optometrists and ophthalmologists in Tanzania, from secondary to tertiary levels of the health care system, have reported a significant increase in the number of patients presenting with KC symptoms. However, little is known about how KC is diagnosed and managed at the different hospital levels, or the protocols and guidelines used across facilities to manage affected patients. Therefore, the purpose of this study, conducted in the Kilimanjaro region, northern Tanzania, was to assess the capacity of the health facilities, and the self-reported ability of the optometrists, to diagnose and manage KC.

## Materials and methods

Applying a mixed-method approach, across-sectional, descriptive study was conducted in July 2024. Using a purposive sampling technique, all health facilities providing eye care services at different levels (from secondary/district level to tertiary) with in the region were included to ascertain the required study data. A quantitative approach, applying the adapted WHO Service Availability and Readiness Assessment (SARA) tool [35], was utilized to gather operational data on the delivery of eye care services specifically related to KC diagnosis and management. The SARA tool was personally administered by the researcher to heads of the respective eye department/unit and, in the absence of the head of the eye department/unit, to the medical officer in-charge or the hospital secretary. Indicators used to assess service availability was the presence of the following: working landline telephone, working computer (desktop/laptop), availability of reliable internet, mobile phone, and reliable electricity. Readiness to provide KC services was assessed based on availability of fourtracer items, namely, basic amenities, diagnostic equipment, staff and guidelines, and services and supplies.

The qualitative component comprised of in-depth interviews with optometrists at selected health facilities for the purpose of investigating their KC knowledge and the diagnostic and management modalities used at their respective facilities. Optometrists were recruited through a combination of telephone calls, WhatsApp messages, and in-person visits. Interviewees were those with a working experience of at least one year because it is expected that during this period they could have encountered, diagnosed and treated KC cases. During recruitment, the optometrists were given information about the study purpose, benefits. risks and participant confidentiality. The study sites, dates and times for the in-depth interviews were agreed upon during the recruitment process. The researcher conducted and recorded the in-depth interviews with the consenting optometrists on the scheduled days. A pre-piloted interview guide was used to collect information on optometrists’ awareness and knowledge of KC and the protocols and modalities used in the diagnosis and management of their respective KC patients.

Quantitative data was analyzed using Statistical Package for Social Sciences (SPSS) version 23.0 and summarized using descriptive statistics. The mean scores for the availability of tracer items and service readiness domains were calculated to obtain the overall mean scores of KC service availability and readiness. Recorded qualitative data was initially transcribed and thereafter, transcripts underwent multiple readings by the researcher for data familiarization and to ensure meaningful descriptions. Data was coded and subsequent thematic and content analysisof the interview data undertaken to extract key concepts and derive common themes.

Ethical approval to conduct the research was secured from the Biomedical Research Ethics Committee of the University of KwaZulu Natal (BREC) with permit number BREC 00007263/2024, and the Department of Research at the Kilimanjaro Christian Medical University College (KCMUCo) with permit no. CREC 2401. Informed consent to participate in the study was obtained from all interviewees and ophthalmologist or heads of the department/unitcompleting the health facility surveys.

## Results

### Health facilities included in the study

Nine health facilities providing eye care services in Kilimanjaro region were included in the study; comprising of one tertiary/teaching hospital, one regional referral hospital, one private hospital and six district level hospitals. Of these, five are owned by faith-based organizations (FBO) and three by the government. All FBO hospitals included in this study are designated council hospitals by the government.

### Quantitative component

#### Characteristics of respondents

Among the nine respondents, the majority were female (56%) with most (44%) aged 50 years or older and having more than 15 years working experience. Of the five heads ofthe eye department/unit, three were ophthalmologists (either specialist or general), one an AMOO and the other an optometrist. Other respondents were two medical officers who were in-charge of the respective health facility whilst two were hospital head secretaries with leadership authority (Table 1).

**Table 1:** Distribution of demographic characteristics of respondents

Variable	Frequency	Percent
*Gender*		
Male	4	44
Female	5	56
*Age group (years)*		
30–39	3	33
40–49	2	22
50 and above	4	44
*Designation*		
Head of eyedepartment	5	56
Medical officer in-charge	2	22
Health secretary	2	22
*Years of work experience*		
1–5	2	22
6–10	2	22
11–15	1	12
More than 15	4	44

#### Service availability and readiness for the provision eye services for KC

Table 2 shows the profile of different professionals available to provide eye care services at the surveyed health facilities. The majority of the hospitals at the different health facility levels had general nurses (100%) and ophthalmic nurses (78%), with most of the eye care professionals being clustered at the referral/consultant hospital (KCMC).

**Table 2:** Profile of professionals employed at eye departments/units

Health facility	Managing authority	Category of health worker						
		Ophthal.(specialist)	General ophthal	AMOO	Optometrist	Ophth. nurse	General nurse	CHW
KCMC T/TH	FBO	2	8	1	6	3	23	1
Mawenzi RRH	Govt	0	1	2	2	1	1	1
St. Joseph DDH	FBO	0	0	0	0	1	1	0
Kibosho DDH	FBO	0	1	1	2	0	4	2
Kilema DDH	FBO	0	0	0	0	2	4	0
Huruma DDH	FBO	0	0	0	0	1	3	1
Hai D/CH	Govt	0	1	0	0	2	2	0
Same D/CH	Govt	0	0	0	0	1	1	0
Kilimanjaro	Private	0	0	0	1	0	6	0
Total staff		2	11	4	10	11	39	5
Percentage of HF with professional		11.1	44.4	33.3	44.4	77.8	100	44.4

*Ophthal* = ophthalmologist; *Ophth* = ophthalmic, *AMOO* = assistant medical officer in ophthalmology; *CHW* = community health worker; *RRH* = regional referral hospital; *T/TH* = tertiary/teaching hospital; *DDH* = designated district hospital; *DH* = district/council hospital; *FBO* = faith-based organization; *Govt* = government; *HF* = health facility; *Qual.* = qualification

#### General availability and readiness of health facilities for provision of KC services

Readiness to provide KC eye care services wasassessed based on availability of fourtracer items as shown in Table 3.Applying the SARA criteriato all facilities surveyed, it was found that only four of the nine surveyed healthcare facilities had all availability tracer itemsfor eye care services for KC patients, securing an overall mean availability score of 4 (44%).

**Table 3:** Tracer item domains for service readiness to provide KC services

HF code	Basic amenities			Diagnostic equipment										Staff and guidelines				Services and supplies				
	Electricity	Communication equipment	Computer with Internet	Refractometer	Keratometer	Slit lamp	Pachymeter	Corneal topographer	Orbscan	B-scan	OCT	Loupe	Lens adjustment equipment	Ophthalmologist	Optometrist	Cornea specialist	Guidelines	Spectacles	Corneal grafting	Cross-linking	Contact lens	Dry eye medication
1	1	1	1	1	1	1	1	1	1	1	1	1	1	1	1	1	1	1	1	1	1	1
2	1	0	1	1	1	1	0	0	0	1	0	1	1	1	1	0	1	1	0	0	0	1
3	1	1	1	1	1	1	0	0	0	0	1	1	0	0	0	0	0	1	0	0	0	1
4	1	1	1	1	0	1	0	0	0	0	0	0	0	1	1	0	0	0	0	0	0	0
5	1	1	1	1	0	1	0	0	0	0	0	1	0	0	0	0	0	1	0	0	0	0
6	1	0	1	0	1	1	1	0	0	0	0	1	0	0	0	0	0	0	0	0	0	0
7	1	0	1	0	0	0	0	0	0	0	0	0	0	0	0	0	0	0	0	0	0	0
8	1	0	1	0	0	0	0	0	0	0	0	1	0	1	0	0	0	0	0	0	0	0
9	1	1	1	1	1	1	0	1	0	0	0	1	0	0	1	0	0	1	0	0	1	1
Total number of HF	9	5	9	6	5	7	2	2	1	2	2	7	2	4	4	1	2	5	1	1	2	4

*1* = available; *0* = not available; *HF* = health facility code; *OCT* = ocular coherent tomography

**Fig. 1:**
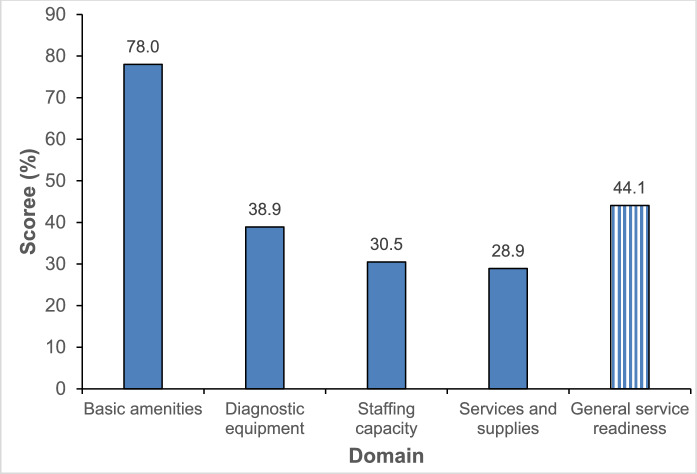
Service readiness index for the management of keratoconus across all facilities

Overall, the mean readiness score to provide basic KC eye care services across healthcare facilities was 44%, with the highest readiness score being demonstrated in basic amenities, available at 78% of facilities and the lowest in services and supplies (29%) (Fig. 1).

### Qualitative component

#### Characteristics of respondents

Ten optometrists participated in the qualitative study component through in-depth interviews. Table 3 shows the characteristics of the participants. Professionally, the majority (70.0%) were diploma holders in optometry with 60% of them having work experience ranging from 11–20 years.

#### Knowledge on keratoconus symptoms and signs

All participants reported being aware of KC as a disease, having been exposed to the condition through formal education or having encountered KC patients in their clinical work settings. Most participants mentioned vision problems (blurred vision, loss of vision), and sensitivity to light (photophobia) as known symptoms of KC. Others added eye rubbing, double vision, myopia, night vision, and halos around lights. The most commonly reported sign of KC was corneal protrusion with a few participants adding corneal scars and allergic conjunctivitis. Responses indicate that participants had a basic knowledge of symptoms, with very limited knowledge of signs of KC, as evidenced in the narrations of two participants below:

*“The symptoms that I know are blurred vision, double vision, sensitivity to light and glare, difficult in night vision and halos around the sight lights.”* [TZ 04]

*“The common sign isprotrusion of the cornea.”* [TZ 05]

#### Risk factors of keratoconus

Responses to questions on KC risk factors revealed a mix of accurate knowledge [TZ 01] with some responses highlighting misconceptions about the disease risks [TZ03]. The accurate and frequently mentioned risk factors included allergies, eye rubbing and vernal keratoconjunctivitis (VKC).

*“Risk factors I know of include severe allergies, antiglare, allergy not treated and VKC”* [TZ 01]

*“Risk factors for this include VKC and atopic keratoconus (AKC).”* [TZ 03]

Further, some participants mentioned low vision and “blind eyes” as risk factors., whilst others said vision loss or blindness.

#### Diagnosis of keratoconus and availability of diagnostic equipment

All participants affirmed that they have diagnosed KC at their clinical settings, reflecting practical experience with the condition. Visual acuity testing, refraction, slit lamp examination, retinoscopy and keratometry were tests mostly performed in the diagnosis of KC.

“*The tests/investigations done here are refraction, retinoscopy, Orbscan, keratometer and slit lamp examination.*” [TZ 06, TZ 03]

While participants at district/designated hospital level depend on Munson’s sign, irregular astigmatism by retinoscope and keratometer readings for diagnosing KC, at the tertiary level health facility, participants reported using an auto/keratometer for refractive errors, tomographers for anterior and posterior corneal mapping and autorefractors for children. Overall, while there is substantial overlap in the basictests performed, the inclusion of specific diagnostic tools and methods varies, indicating diverse approaches to ocular assessment across different facility levels.

#### Management of keratoconus

The management procedures described by participants demonstrate both commonalities and differences in how facilities manage KC and refractive errors. Most participants reported that their main management approach is to provide corrective optical devices, with spectacles prescribed as the primary means to correct vision and rigid contact lenses being used for more advanced stages of KC. These practices reflect a shared focus on managing visual impairment through optical aids. Participants were aware of other non-optical treatment modalities utilized at their facilities or at other referral facilities as indicated in quotes below:

*“We do provide spectacles and contact lens when there is little progression. Cross-linking, though not done here, is suggested for referrals and Vitamin B is prescribed to improve or reduce the progression of keratoconus.”* [TZ 05].

*“We perform correction of refractive errors by using spectacles at early stages. At progression stages, contact lenses are used, depending onthe stage of disease progressionwith corneal transplants at very late stages.” [TZ 10].*

Participant responses highlight different approaches applied for advanced KC treatment protocols. At lower level facilities treatment mainly involves provision of spectacles and RGP lenses and referrals for other required procedures. At the tertiary level, patients are managed with surgical interventions such as CXL and corneal transplants. Participants from lower-level healthcare facilities indicated that KC patients are referred to specialist ophthalmologists at tertiary facilities, as treatments such as corneal cross-linking and keratoplasty are beyond the respective facility's capacity. They emphasized that having to refer patients to ophthalmologists or specialised centers like KCMC, makes them reliant on external resources for comprehensive KC patient management.

#### Suggestions for improvement of KC diagnosis and management

##### Training and continuing education for KC management

Several participants highlighted the need for increased training and education opportunities to improve their knowledge and skills in KC management. Participants underscored the importance of training of all practitioners, noting specific needs in corneal transplant surgery for ophthalmologists and contact lens fitting for optometrists. They suggested including all optometrists in KC training initiatives to increase the number of facilities with expertise available for managing KC. The additional training will aid to improve skills on early diagnosis and management of early KC as well as to appropriately identify cases to refer for advanced management of KC.

Further, participants strongly recommended employing more specialists, such ascorneal specialists; and emphasized that having sufficient numbers of persons with specialised knowledge will help them to comprehensively manage KC patients at their respective facilities. A few suggested that the more experienced and trained practitioners should be willing to impart clinical skills and KC management models to newer graduates. Others called for advocacy programmes to increase awareness among staff and patients as raised by participant TZ02:

*“Awareness creation to patients is needed to inform them that KC is a treatable condition and to encourage them to attend healthcare facilities early.*” [TZ 02].

There was a strong collective consensus on the need for advanced diagnostic equipment and adequate clinical resources, such as trial contact lenses. Raising a lack of equipment and inadequate facility support in acquiring equipment as barriers, all participants emphasized the importance of procuring and making available appropriate diagnostic tools and equipment at all facilities. Additional barriers raised included deficiencies in the KC training in undergraduate programmes and insufficient continuing professional development (CPD) programmes designed to assist practitioners to keep abreast with technological advancements as cited below:

*“Equipment for KC are few, also we lack contact lenses and, at school we werenot taught about contact lenses.”* [TZ 09].

*“Maybe we should do CPD programmes on contact lens management.”* [TZ 03].

#### Outreach and screening services for keratoconus

Identifying the need for early detection and intervention, which can help in managing the condition more effectively, a few participants suggested improving outreach and KC screening services at their respective facilities, to reach children at younger ages.

*“Keratoconus is only seen at the clinic so there is a need to improve outreach and screening of KC at young ages, also training more people on KC skills.”* [TZ 10].

## Discussion

The purpose of the study was to conduct an evaluation at health facilities in Kilimanjaro region, northern Tanzania to assess the availability of KC services and capacity of the facilities to diagnose and manage KC. Additionally, the study investigatedthe self-reported capacity of optometriststo effectively diagnose and manage KC. Engagement with facility practitioners is critical as they are key stakeholders in eye care service delivery. Through reporting on their daily lived experiences and reflecting on their individual knowledge and skills, they are able to provide valuable insights into the overall delivery of KC services within the facility and region.

### Service availability and readiness

Service availability refers to the physical evidenceof the delivery of services and encompasses health infrastructure and core health personnel[36]. Service-specific readiness refers to the ability of health facilities within a specified area (region, zone or country as a whole) to offer a specific service, and the capacity to provide that service measured through consideration of tracer items. These include trained staff, guidelines, equipment, diagnostic capacity, and medicines and commodities[37] Results revealed that less than half (44%)of the health facilities providing eye care services in Kilimanjaro region have KC services available to be in a state of readiness to provide KC services. Cited reasons for the low level of service availability and readiness for KC, confirmed through the questionnaire data and participant feedback, were the shortage of qualified eye care personnel, inadequate practitioner knowledge of the disease and a lack of relevant diagnostic equipment at the facilities. Fumbwe et al., (2021) conducted a service availability and readiness study in Tanzania and found the mean facility availability of general eye care services to be 46%, with only 2% of the facilities having all the required tracer items for eye care provision. The lack of general eye care services potentially implies that service availability and readiness for KC could be at an even lower level nationally than found in this study in the Kilimanjaro region. The health care infrastructure and services in Kilimanjaro is more developed than other regions as it includes a number of consultant, referral and FBO hospitals that provide eye care services. The deficiencies in KC care revealed in 56% of the facilities in Kilimanjaro implies that patients afflicted with KC are likely to experience delayed diagnosis and management if services are not available at their local health facility, increased costs to travel to facilities further away and an overall prolonged negative impact on patient’s well-being and quality of life. Noting the global call for universal health coverage, it is recommended that health care leadership in the country consider the study results in future health services planning.

### Optometrists’ knowledge of KC and awareness of risk factors

Despite all participants reporting that they were aware of KC, the varying, and overall limited, knowledge of signs, symptomsand risk factors revealedraises a serious concern.This could be attributed to the majority of participants reporting that they had learnt about KC during their undergraduate studies, which in most instances was completed ≥ 10 years ago (Table 1 & 3).Further, older optometrists, who are diploma holders, reported not being exposed to KC during their training, as it was not incorporated into the old curriculum (Personal communication with the Principal, School of Optometry at KCMC, August, 2024). Reinforcing the impact of poor training, was the admittance by some optometrists that, despite having the required equipment and associated resources at their respective work facilities, they were not adequately competent in the use of the equipment to diagnose and manage KC. This highlights a gap in undergraduate education in KC and insufficient work-based training on equipment and clinical diagnostic tools procured by facilities [38]. Exacerbating the situation is the lack of continuing professional development (CPD) programmes that could enhance their knowledge and skills in KC diagnosis and management. In planning CPD programmes there should be training in KC, and companies from whom equipment is purchased should be approached to conduct training sessions for practitioners to ensure ease of use by all.

Several studies conducted in sub-Saharan Africa have reported similar deficiencies in optometrists’ knowledge on KC [39, 40]. This highlighted knowledge gap across the sub-Saharan region could be addressed through a collective response. Experienced individuals should be approached to undertake activities to share knowledge and up-skill practitioners in KC. Presentations and wet-labs on KC could also be incorporated into regional conferences, which may have a wider reach than local CPD activities and create access where skilled practitioners are not readily available.

### Diagnosis and management of keratoconus

Apart from needing knowledgeable optometrists, early detection of KC requires the availability of appropriate diagnostic equipment. The lack of required equipment for KC diagnosis and management at many of the facilities, alsoreported elsewhere in Africa [39, 41, 42], hinders practitioners from assisting presenting patients. At district/designated hospital level clinicians depend on Munson’s sign and irregular astigmatism, derived from a retinoscope and keratometer to diagnose KC, and at tertiary level, more advanced diagnostic equipment such as an ocular corneal topographer, tomographer and autokeratometer are used.

This current model is not appropriate for early detection and the lack of diagnostic capability at district level leads to Munson’s sign, which manifests at an advanced stage of KC, being used primarily in diagnosis. This lack of early detection in lower level facilitiesis generally too late and denies the patient the opportunity for early intervention procedures such as corneal cross-linking (CXL) to be performed. Apart from affecting the patients QoL, late diagnosis is also not cost-effective; as the costs of managing advanced KC is higher than that needed for early KC [7, 38, 39, 43]. It is recommended that the government (responsible ministry) develop a protocol to enable early detection and management on site for timely referral to higher level referral centers.

Thestudy revealed that, at early stages of the condition, the majority of KC patients were only offered spectacles as a treatment option, and soft or hard contact lenses being prescribed at late stages. Of the nine facilities visited, only two hospitals dispense contact lenses for KC patients (Table 4), a situation raising patient care concerns. Sufficient evidence exists that spectacles may only be useful in the early stages of KC [44]. Additionally, the majority of KC patients require RGP corneal or scleral lenses as optical device interventions and cross-linking, intrastromal rings or keratoplasty as surgical interventions [45, 46]. Implications for not providing appropriate KC treatment can result in severe vision impairment with the associated psychological and social impact [47]. A possible reason for a lack of appropriate treatment options could be linked to a lack of knowledge of facility practitioners on the most appropriate treatment modalities for the various disease stages or, challenges with procurement of other corrective devices. To ensure acceptable standards of care, all facilities, either on site or through referrals, should ideally offer patient education, spectacles, contact lenses, CXL and surgical options to KC patients. The study findings align with those reported in a West African study where 77.7% reported not fitting contact lenses and 68% were willing to refer the patients to another practitioner, while the major barrier reported was alack of experience [48]. This study, combined with previous findings on the continent [37, 49, 50] reiterate the call for initiatives to train practitioners in Africa to better manage KC patients and provision of appropriate treatment modalities.

**Table 4:** Employment characteristics of the participants interviewed (n = 10)

Variable	Frequency	Percent
*Facility employed at*		
KCMC Zonal Hospital	5	50
Mawenzi Regional Referral Hospital	2	20
Kibosho Designated Council Hospital	2	20
Kilimanjaro District Hospital	1	10
*Level of facility*		
Consultant/Tertiary level	5	50
Regional (referral level)	2	20
District	3	30
*Facility location*		
Moshi Urban	8	80
Moshi Rural	2	20
*Professional qualification*		
BSc. Optometry	3	30
Diploma in optometry	7	70
*Years of experience*		
1–10	2	20
11–20	6	60
Above 20	2	20

The high cost of diagnostic equipment was highlighted as a contributing to the lack of equipment, ultimately impacting the ability to diagnose and manage KC. Similar studies in other African countries, including Kenya, Cameroon, Ghana, Uganda and South Africa have reported that access to affordable equipment for KC diagnosis and treatment remains a persistent challenge [34, 51]. Advanced diagnostic equipment has the capacity to detect very early KC and promote early interventions, which significantly reduces long term patient management costs. It may be prudent for optical equipment manufacturers to collaborate and innovate to develop more cost-effective equipment that countries in Africa, with limited health budgets, can afford. Investment in diagnostic equipment will help to reduce the higher and long-term costs of treatment for advanced KC, such as corneal transplants.

The complete package of KC services was available at only one tertiary hospital (Table 4) in the northern zone, which serves a population of approximately 15 million people [52]. This finding may expose inadequate facility and service distribution planning. Attention should be given to structuring eye care services, encompassing different levels of care, to ensures easy access to appropriate, quality eye care services for the country. Having only one tertiary hospital for 15 million people may explain the long waiting times often experienced by patients. Although some optometrists raised as a problem that referring patients to tertiary specialized centers makes them reliant on outside resources, this system of referral should be encouraged, as it is not feasible that the same level of care be offered from primary to tertiary level. However, it highlights that a coherent referral system is required and communicated to all eye care workers within the system to enable understanding.

Advocacy efforts are needed for hospital management and policymakers to understand the importance of comprehensively addressing KC, understanding key aspects of the disease and its impact on the individual, community and government resources. It will further promote adequate allocations of funding to train practitioners, and procure appropriate equipment and other clinical resources for comprehensive management of KC. Prioritizing KC treatment in healthcare planning would improve patient outcomes, which could lead to significant long-term savings and improve QoLof the patients.

### Limitations

The findings of this study are not devoid of limitations. A potential study weakness is that it included healthcare facilities in only one regionin Tanzania, although it is the most populous region in term of medical services in the country. There may be a possibility that practitioners preferred not to declare personal knowledge and skills shortcomings, however, the responses from this cohort was sufficient to determine general trends as evidence of the shortcomings of the collective. A recommendation is for there to be a larger nationwide study to elucidate the current situation of service availability and readiness for the diagnosis and management of KC in the country.

## Conclusions

The study findings indicate both a low service availability and service readiness for provision of KC eye care services. This couldbe attributed to a lack of availability of KC services in the majority of facilities in the region, shortage of appropriate diagnostic equipment, inadequate practitioner knowledge on KC and unavailability of required diagnostic and treatment supplies, such as contact lenses. To facilitate early KC detection, diagnosis and management there is need to equip lower-level health facilities with adequate qualified personnel, equipment and resources for KC management. Moreover, regular outreach services such as school screening programs for refractive errors should be initiated and/or strengthened in order to reach undiagnosed persons.

Healthcare leadership in Kilimanjaro and Tanzania should reflect on the shortcomings highlighted in this study in future eye care service planning. Measures should be taken to engage in activities to amend policy, devise multi-level eye care system planning, which includes adequate human resource compliments and appropriate funding allocations for equipment and supplies, for comprehensive service delivery. Further, practitioners must be re-trained in KC diagnosis and management to foster quality care in patient management.

### Strength of the study

To the best of our knowledge, this is the first study in the country that dealt with service availability and readiness assessment, specifically for KC. It couldserve as a catalyst for larger studies for this condition that affects mostly the school-going children and young adults and hence hinders their academic and occupational performance, negatively affecting their quality of life.

## Data Availability

No datasets were generated or analysed during the current study.
